# Are classification criteria for vasculitis useful in clinical practice? Observations and lessons from Colombia

**DOI:** 10.1186/1740-2557-6-1

**Published:** 2009-02-27

**Authors:** Paúl Alejandro Méndez Patarroyo, José Félix Restrepo, Samanda Adriana Rojas, Federico Rondón, Eric L Matteson, Antonio Iglesias-Gamarra

**Affiliations:** 1 Rheumatology Fellow, Department of Internal Medicine, Rheumatology Unit, Universidad Nacional de Colombia, Bogota, Columbia; 2 Professor of Medicine, Department of Internal Medicine, Rheumatology Unit, Chief of Rheumatology Unit, Universidad Nacional de Colombia, Bogota, Columbia; 3 Assistant Professor, Department of Internal Medicine, Rheumatology Unit, Universidad Nacional de Colombia, Bogota, Colombia; 4 Professor of Medicine, Mayo Clinic College of Medicine, Rochester, Minnesota, USA; 5 Professor of Medicine, Department of Internal Medicine, Rheumatology Unit, Universidad Nacional de Colombia, Bogota, Colombia

## Abstract

**Introduction:**

Idiopathic systemic vasculitis represents a group of clinical entities having non-specific etiology with the common characteristic of acute or chronic inflammatory compromise of the small and large vessels walls, associated with fibrinoid necrosis.

**Objectives:**

To describe the most common inflammatory vascular diseases in a long historical cohort of patients from San Juan de Dios Hospital located in Bogota, Colombia using two different systems and a clinical histopathological correlation format, and to make a comparison between them.

**Methods:**

We reviewed all previously ascertained cases of vasculitis confirmed by biopsy processed between 1953 and 1990, and systematically collected data on all new cases of vasculitis from 1991 to 1997 at the Hospital San Juan de Dios (Bogota – Colombia). The cases were classified in accordance with the Chapel Hill Consensus criteria, and the system proposed by J.T. Lie.

**Results:**

Of 165,556 biopsy tissue specimens obtained during this period from our hospital, 0.18% had vasculitis, perivasculitis or vasculopathy. These included 304 histopathological biopsies from 292 patients. Cutaneous leukocytoclastic vasculitis (64 histological specimens) was the most frequently encountered type of "primary" vasculitis followed by thromboangiitis obliterans (38 specimens), and polyarteritis nodosa (24 specimens). Vasculitis associated with connective tissue diseases (33 specimens) and infection (20 specimens) were the main forms of secondary vasculitis, a category that was omitted from the Chapel Hill consensus report. We found that 65.8% of our histopathological diagnoses could not be classified according to the Chapel Hill classification, and 35.2% could not be classified according to the classification of Lie. Only 8.9% of cases remained unclassified by our system after clinical and histological correlation.

**Conclusion:**

Current vasculitis classification schemes are designed for classification, rather that diagnosis of disease and do not adequately address some common forms of inflammatory vascular diseases, including those of infectious etiology and unusual etiology seen in clinical practice. Based on our clinical experience, we suggest a classification outline which practitioners can use which emphasizes correlation of the clinical picture to the histopathology findings for diagnosis and therapy, which may promote better clinical practice and standardization for clinical trials.

## Introduction

Systemic vasculitis represents a heterogeneous group of clinical entities having non-specific etiology with the common characteristic of acute or chronic inflammatory compromise of the small and large vessels walls, associated with fibrinoid necrosis. Histopathological examination is the main basis for diagnosing these diseases. Vasculitis may be primary, or associated with another diseases process. Infections, hypersensibility to drugs, atrial myxoma, solid cancers, and lymphoproliferative or other connective tissue diseases are among the entities causing secondary vasculitis [1-17].

Criteria have been defined for classifying the different types of vasculitis by the American College of Rheumatology (ACR) Vasculitis Study Committee, which however limited itself to defining "primary" vasculitis characteristics. The ACR introduced criteria for some vasculitides in 1990, and has proposed different parameters for classifying each one of these entities [18-26]. Only seven types of vasculitis are considered in the ACR scheme. These and the classification system proposed by J. T. Lie [27] and by Chapel Hill consensus conference [28] in 1994 are in use today. These criteria are not designed for diagnosis of vasculitis, and are inadequate for clinical application, and omit important clinical forms of vasculitis.

With few exceptions, only sporadic reports of vasculitis are found in Latin-American literature [29-56]. Some of the larger series include classification studies of Alarcón-Segovia [57-59] and one cohort of patients with primary vasculitis of the Cisterna's group in Chile [60]. The epidemiology of the primary vasculitides is best studied in Europe and North America [61-72]. There have been no such studies in South America.

With this study, we describe the different types of vasculitis most frequently found in the San Juan de Dios' Hospital from Bogotá, Colombia using two current classification systems as well as our own clinical-pathological correlation.

## Methods

This is a descriptive study performed in two phases. All 165,556 histological specimens (129,192 between 1953 and 1990 and 36,364 between 1991 and 1997) processed in the Universidad Nacional de Colombia's Pathology Department (Bogotá, Colombia) were compiled. The data from the 1953–1990 cases were obtained from medical record review of all patients with a positive biopsy specimen. After 1990, cases were compiled using a standard case report form (CRF) as patients attended consultation in the clinical setting. All reports from each examination were reviewed and all those with vascular inflammatory compromise collected; 304 confirmed cases of vasculitis were found (187 between 1953 and 1990, and 117 between 1991 and 1997). These included 187 specimens from the 1953–1990 period and 117 from 1991–1997. These specimens were reviewed by rheumatologists and by members of the pathology department of a third level university hospital from Bogotá, Colombia (Hospital San Juan de Dios).

Results were expressed in frequencies since the total number of patients that consulted during these years was not available, so that an approximate calculation of incidence could not be performed. Of 165,556 biopsies processed by the histopathology laboratory of our hospital, 304 specimens from 292 patients had evidence of vascular inflammatory involvement. Once a diagnosis was made on histopathological grounds, the cases were classified according to the Chapel Hill [28] and J. T. Lie classification proposal [27]. The criteria defined by the ACR [18-26] for classifying primary vasculitis were also applied, however these encompass only 7 types of vasculitis, and it was necessary to expand the spectrum of vasculitis entities according to clinical, laboratory, and histological considerations. All histological studies were re-reviewed and diagnosed according to clinical, laboratory and histopathological information.

## Results

Of the 165,556 tissue specimens, 0.18 percent could be assigned a diagnosis of vasculitis, perivasculitis or vasculopathy (Tables 1 and 2). Three-hundred-four histopathological biopsies from 292 patients with documented vasculitis were found during that period. More than one sample was obtained from twelve patients, those with two histological specimens were: six with Buerger's disease (in one of these the diagnosis on first biopsy was not conclusive); two patients with polyarteritis nodosa (in both the initial diagnosis was inconclusive); two patients with inconclusive results of histology, including one diagnosed with cutaneous leukocytoclastic vasculitis and the other with connective tissue disease vasculitis.

**Table 1:** Classification of histological specimens with vasculitis from the San Juan de Dios Hospital, Bogotá, Colombia, 1953 – 1997, according to the Chapel Hill system [28]

**Vasculitis diagnosis**	**Number** N = 304	**Frequencies** per 100,000 histological specimens	**Percentages**	
			
			groups of vasculitis	subgroups of vasculitis
**I. Primary vasculitis**	**104**	**62.8**	**34.2**	

A. Large vessel vasculitis	1	0.6		1

1. Giant cell (Temporal) arteritis	1	0.6		

B. Medium sized vessel vasculitis	24	14.5		23.1

1. Polyarteritis nodosa	24	14.5		

C. Small vessel vasculitis	79	47.7		75.9

1. Wegener's granulomatosis	3	1.8		

2. Churg-Strauss syndrome	2	1.2		

3. Microscopic polyangiitis	1	0.6		

4. Henoch-Schönlein purpura	9	5.4		

5. Cutaneous leukocytoclastic vasculitis	64	38.7		

**II. Not classified vasculitis**	**200**	**120.8**	**65.8**	

Total	304	183.6	100	

**Table 2:** Classification of histological specimens with vasculitis from the San Juan de Dios Hospital, Bogotá, Colombia, 1953 – 1997, according to the scheme of Lie [27]

**Vasculitis diagnosis**	**Number** N = 304	**Frequencies** per 100,000 histological specimens	**Percentages**	
			
			groups of vasculitis	subgroups of vasculitis
**I. Primary vasculitis**	**142**	**74**	**46.7**	

A. Affecting large, medium and small sized blood vessels	1	0.6		0.7

1. Giant cell (Temporal) arteritis	1	0.6		

B. Affecting predominantly medium and small-sized blood vessels	29	17.5		20.4

1. Polyarteritis nodosa	24	14.5		

2. Churg-Strauss syndrome	2	1.2		

3. Wegener's granulomatosis	3	1.8		

C. Affecting predominantly small-sized blood vessels	74	44.7		52.1

1. Microscopic polyangiitis	1	0.6		

2. Henoch-Schönlein purpura	9	5.4		

3. Cutaneous leukocytoclastic angiitis	64	38.7		

D. Miscellaneous conditions	38	23		26.8

1. Buerger's disease	38	23		

**II. Secondary vasculitides**	**55**	**33.2**	**18.1**	

1. Infection-related vasculitis	20	12.1		

2. Connective tissue disease vasculitis	33	20		

3. Hypocomplementemic urticarial vasculitis	1	0.6		

4. Vasculitis associated with neoplasia	1	0.6		

**III. Unclassified vasculitis**	**107**	**64.6**	**35.2**	

Total	304	183.6	100	

The average age of the patients from whom these specimens were obtained was 36 years, ranging from 10 to 99. Male/female ratio was 1:2. Most specimens corresponding to vasculitis came from skin (64.5%), amputated extremities (11.5%) and muscle (8.2%).

Primary vasculitis was the diagnosis in the minority of our biopsies; thirty-four percent (104 specimens) of the histopathological studies corresponded to primary vasculitis according to the Chapel Hill Consensus Conference while forty-six percent (142 specimens) revealed a primary vasculitis according to the system of J. T. Lie (Tables 1 and 2).

The most frequently encountered types of primary vasculitis were cutaneous leukocytoclastic vasculitis (64 specimens), followed by thromboangiitis obliterans (38 specimens) (all of this obtained from tissue of amputated extremities), polyarteritis nodosa (24 specimens), and Henoch-Schönlein purpura (Tables 1, 2, 3).

**Table 3:** Histological specimens with vasculitis from the San Juan de Dios Hospital, Bogotá, Colombia, 1953 – 1997, according to a clinical-pathological correlation of vasculitis [92]

**Vasculitis diagnosis**	**Number** N = 304	**Frequencies** per 100,000 histological specimens	**Percentages**	
			
			groups of vasculitis	subgroups of vasculitis
**1. Leukocytoclastic vasculitis**	**107**	**64.6**	**35.2**	

1.1. Erythema elevatum diutinum	1	0.6		0.9

1.2. Secondary to diseases	42	25.4		39.3

1.2.1. SLE	14	8.5		

1.2.2. PM/DM	14	8.5		

1.2.3. Diffuse/limited scleroderma	5	6.7		

1.2.4. Henoch-Schönlein purpura	9	5.4		

1.3 Livedoid leukocytoclastic vasculitis	5	3		4.7

1.4. Idiopathic	59	35.6		55.1

**2. Lymphomonocitic's vasculitis**	**53**	**32**	**17.4**	

2.1. Schamberg's purpura	1	0,6		1.9

2.2. Chronic urticaria, associated	1	0.6		1.9

2.3. Idiopathic	51	30.8		96.2

**3. Nodular vasculitis**	**19**	**11.5**	**3.6**	

3.1. Erythema nodosum	7	4.2		36.8

3.2. Granulomatous panniculitis	2	1.2		10.5

3.3. Vasculitis in panniculitis	4	2.4		21.1

3.4. Idiopathic	6	3.6		31.6

**4. Granulomatous vasculitis**	**11**	**6.6**	**3.6**	

4.1. Wegener's granulomatosis	3	1.8		27.3

4.2. Churg Strauss	2	1.2		18.2

4.3. Lymphomatoid granulomatosis (lung)	1	0.6		9.1

4.4. Granuloma annulare	1	0.6		9.1

4.5. Giant cell (Temporal) arteritis	1	0.6		9.1

4.6. Idiopathic	3	1.8		27.3

**5. Polyarteritis nodosa**	**24**	**14.5**	**7.9**	

5.1. Generalized	16	4.8		66.7

5.2. Cutaneous	8	9.7		33.3

**6. Vasculitis like diseases**	**4**	**2.4**	**1.3**	

6.1. Arterio-embolism	4	2.4		100

**7. Infection-related vasculitis**	**20**	**12.1**	**6.6**	

7.1. Mycobacterium	12	7.2		60

7.2. Bacterial infections	8	4.8		40

**8. Microscopic polyangiitis**	**1**	**0.6**	**0.3**	

**9. Thromboangiitis obliterans**	**38**	**23**	**12.5**	

**10. Unclassified vasculitis**	**27**	**16.3**	**8.9**	

**TOTAL**	**304**		**100**	

Vasculitis associated with connective tissue diseases (33 specimens) was the most frequently encountered classification among those specimens classified as secondary vasculitis according to the scheme proposed by J.T. Lie, while vasculitis associated with infection was the next most common type (20 specimens; Tables 2 and 3).

In our format of clinical-pathological correlation we found that leukocytoclastic vasculitis was the most frequent with 107 specimens, followed by lymphomonocytic vasculitis with 53 specimens. Only 27 specimens (8.9%) could not be classified with our or any scheme. Within the cases that were unable to be classified using the system of JT Lie or the Chapel Hill proposal are the lymphomonocytic vasculitis (53 specimens), several cases of granulomatous vasculitis, nodular vasculitis (19 specimens) and illnesses that resemble vasculitis, including arterial embolism (4 specimens) (Table 3).

## Discussion

Vasculitis classification are diverse and employ several organizing principles including the size of the compromised vessel, whether an underlying pathoetiology is found (primary versus secondary vasculitis); the main type of vessel compromise (artery, vein, capillary, etc.), the type of immune damage and others.

Attempts to classify vasculitis began with the work of Pearl Zeek in 1952 [61] and many others have proposed their own classification schemes [27,28,57-59,62-91]. Considerations of the ACR [78], Lie [27,79-81], Churg [83,84], and others were taken into account during development of the Chapel Hill consensus conference criteria [28,91].

Zeek's concept of classifying vasculitis according to the size of the comprised vessel: small, medium-or large-sized continues to be widely used. This proposal, along with other proposals regarding vessel size, has been adapted by many authors who have since built upon it.

Existing classification systems are incomplete and difficult to use in the clinic, as they are not designed to be diagnostic criteria, but rather are more useful in distinguishing one type of vasculitis from another in populations of patients for whom a diagnosis of vasculitis is established [92]. For this reason, in 1993 we proposed a format for ordering vasculitides based on their pathogenesis, histologic features, and causal agents if known, with the intent of improving understanding of them without proposing a new classification scheme (Table 3). We consequently included numerous entities with defined histology which are not captured in existing classification schemes [93]. This provides a clinical-pathologic correlation which goes beyond the incomplete current classification schemes [93].

It is our opinion that the most representative, recognized and utilized classifications in worldwide literature are those of J.T. Lie, and Jennette and Falk *et al*., described at the Chapel Hill Consensus Conference. We therefore analyzed this Colombian cohort in accordance with these two classification schemes and compared them with our findings based on clinical-pathological correlation.

In our experience, "primary" vasculitis seems to be uncommon in Colombia [41], although it does occur, including Takayasu's disease [42,92]. Since our review concentrates on cases with biopsy evidence of disease, the true frequency of primary vasculitis diagnosed on clinical, but not histological grounds, is certainly higher. Furthermore, we observed that a significant number of our cases could not be classified by Lie's and the Chapel Hill Consensus criteria (Table 3, 4). We also encountered difficulties in assigning a diagnosis of vasculitis to some of our histological studies according to whether they were primary or secondary. Many difficulties emerged while evaluating these cases. It was particularly difficult to make a diagnosis based on the ACR diagnostic criteria of classic types, since these were created for classifying patients having defined vasculitis and not for making a specific or even general diagnosis in patients where other vasculitis or vasculopathies are suspected.

**Table 4:** Comparison of Two Vasculitis Classifications Schemes With a Clinical-Pathological Correlation from the San Juan de Dios hospital, Bogotá, Colombia, 1953 – 1997 *

	**Percentages**		
	
	**Chapel Hill**	**Lie**	**Clinical-pathological Correlation**
Classifiable	34.2	64.8	92.1

Unclassifiable	65.8	35.2	8.9

* from 304 histological specimens with inflammatory vascular compromise

The difficulty in applying these criteria clinically was illustrated in a study using chart audits to classify 198 consecutive patients by ACR criteria, with re-audit of patient charts after 2–8 months to ascertain the patient's ultimate diagnosis, Rao et al, found that vasculitis was diagnosed in 51 (26%) patients. Thirty-eight (75%) of 51 patients with vasculitis and 31 (21%) of 147 patients without vasculitis met ACR criteria for one or more types of vasculitis. The positive predictive values for the four vasculitides according to ACR criteria were 17% to 29% for the entire cohort and 29% to 75% for only the patients with a final diagnosis of vasculitis [94]. It was also difficult to obtain samples in cases where visceral compromise or large vessel involvement was present, although clearly the histopathologic examination continues to be the gold-standard for diagnosis.

We have pioneered the study of vasculitis in Colombia. The San Juan de Dios Hospital was the Colombian reference center for this type of pathology during the time of this study, and our reports may reflect the state of these entities in Colombia and perhaps reveal Latin-American trends in frequency of the different types of vasculitis occurring. Small-sized vessel vasculitis is the predominant type of primary vasculitis.

In the Latin American literature most reports of vasculitis are those of idiopathic types. Takayasu's arteritis in Brazil has been reported by Sato et al's group [43], and Behçet's disease by Heyman et al and Ferraz et al [44,46]. Kawasaki's disease has been reported by Saraiva et al [45], and Lucio's phenomenon has been reported by Souza et al [38]. Cisternas et al evaluated described their patient cohort of "primary" vasculitis in Chile [60]. Other work groups in Mexico have made significant contributions to the study of vasculitis, especially that of Alarcón-Segovia. The immuno-pathological aspects of Takayasu's arteritis have been reported on by Vargas-Alarcon [48]. Other studies include cases of Takayasu's arteritis by Castro, Castanon, and Robles [49,53,56]. Henoch-Schönlein purpura has been reported by Reyes-Vasquez [50], Kawasaki's diseases in children by Vizcaino-Alarcon [52], and Behçet's disease by Alarcon-Segovia's [55]. In Peru, Sanchez et al [95] described 29 patients with microscopic polyangiitis and compared them with those of Guillevin's group [96]. The Peruvian patients had more multiple mononeuritis, less cutaneous involvement, more benign renal disease, and a better prognosis. There are no studies of vasculitis epidemiology in Latin American.

Cutaneous leukocytoclastic vasculitis predominated in our study of primary vasculitis correlating with other studies reporting this as being the most frequent type.

Thromboangiitis obliterans was more frequent before 1990 (26 specimens), probably because the national health promotion effort from the 1980s onward against tobacco-use could account for its diminishing frequency since then. The difference found in primary vasculitis between the two proposed classifications is explained largely by the absence of Buerger's disease in the Chapel Hill scheme. Thromboangiitis obliterans (Buerger's disease) is included as a vasculitis, understanding that it is histopathologically distinguished from other forms of vasculitis, having an inflammatory thrombus with relatively sparse evidence of inflammation in the vessel wall.

Our study found JT Lie's classification was better adapted to those pathologies associated with vasculitis in our institution compared to that of the Chapel Hill Consensus Conference (Table 4). This may be due to the consensus excluding secondary vasculitis, such as those associated with connective tissue diseases, solid tumor, atrial myxoma, drug- or infection-related reactions, among others. A majority, 65.8%, of the analyzed specimens were not classified on applying Chapel Hill consensus criteria, while fewer remained unclassified using Lie's proposed criteria (35.2%). When we used a correlation based on clinical diagnosis with pathological findings, only 8,9% of the specimens were unable to be classified.

Histopathologic examination of biopsy specimens is the gold standard for studying vasculitis in spite of existing limitations. Currently used classifications leave many vascular entities, including some forms of vasculitis which are thus far ill-defined, without being classified. Knowledge concerning these entities is limited in Latin America, so efforts made in studying these diseases will benefit our patients. It is also necessary to make extra efforts towards creating more suitable classifications in which Latin-Americans and other countries study groups may participate.

It is likely that the ethnicity, immunogenetics, and the socioeconomic status of patients with vasculitis, among other factors, is different in our patients than in those described in North American and Western European cohorts and accounts for many of the clinical differences seen in these different groups of patients. Consequently, an attempt must be made to include the totality of conditions with clinical and histopathological findings of vasculitis and even vasculopathies mimicking or closely resembling established vasculitis conditions in such new classification schemes to facilitate clinical diagnosis and research.

## Conclusion

Current classification criteria for vasculitis do not adequately address some common forms of inflammatory vascular diseases, including those of infectious etiology and unusual etiology seen in clinical practice. They are based primarily on the vessel size, rather than on histopathologic clinical correlation. There are about 20 forms of primary vasculitis, some of which are not included in the current classification schemes such as nodular vasculitis of the skin, as well as many forms of secondary vasculitis including those associated with neoplasia, infection, and medications, which are not included in current nosology.

The importance of establishing this correlation is illustrated by, for example, the finding of lymphomonocytic vasculitis which is typically associated with cutaneous vasculitis, often but not always related to infection, medication or collagen vascular disease, and typically has a good prognosis. The several forms of ANCA associated vasculitis have pleomorphic manifestations which demand a critical differentiated clinical approach. Based on our clinical experience, we suggest a classification outline which practitioners can use which emphasizes correlation of the clinical picture to the histopathology findings for diagnosis and therapy, which may promote better clinical practice and standardization for clinical trials.

A more complete understanding of these entities may provide a better basis for classifying them. Our study suggests that current classification schemes for vasculitis are incomplete [97]. They must be brought up to date [98], and must better reflect the diagnostic process used clinically. At present, physicians should use the current classification systems with the understanding that they are incomplete, and have limited value in clinical practice [99]. Meanwhile, we think that judicious analysis of cases of suspected vasculitis through clinical-pathological correlation will continue to be important in assessment of patients with these diseases.

## Competing interests

The authors declare that they have no competing interests.

## Authors' contributions

PAM-P participated in the design of the study, acquisition of data, data analysis, and interpretation of the data. J-FR participated in the design of the study, acquisition of data, data analysis, and interpretation of the data and manuscript preparation. SAR participated in the acquisition of data, data analysis, and interpretation of the data. FR-H participated in the design of the study, acquisition of data, data analysis, and interpretation of the data. ELM participated in the data analysis, interpretation of the data, and manuscript preparation. AI-G participated in the design of the study, acquisition of data, data analysis, and interpretation of the data and manuscript preparation. All authors read and approved the final manuscript.
